# 

**Published:** 1898-06

**Authors:** 


### EDITORIAL.

### First Annual Meeting of the National Dental Association.

This meeting will be held on the last Tuesday of August, prox. Omaha, Neb., was selected as the place for the meeting. Upon investigation, however, by the Executive Committee great doubt has arisen as to the feasibility of holding the meeting at that place, because of inadequate accommodation. The statement was made at the meeting last year that ample accommodation would be available at Omaha for the meeting.

Some members of the Executive Committee, however, have visited Omaha and have become strongly impressed that proper accommodations cannot be had, as they state that Omaha is not well supplied with hotels and such as they have are small and very limited in their capacity, and furthermore an Exposition is being held and will continue until autumn. This we know, from personal observation, is a very fine affair, and will undoubtedly draw large numbers of people to it during the coming months. It is thought that the accommodation afforded by Omaha will not more than meet the demand thus created. The attendance at the National Dental Association will undoubtedly be quite large, and it is thought that there will be larger attendance than usual, secured by the reorganization. The National Association of Dental Faculties meets at the same time and place, and will considerably add to the numbers requiring accommodation.

From what we saw in Omaha and could learn, the impression is strong that unless the people of the city open their homes for the accommodation of these societies they will be but poorly provided for. If there is assurance that the families will take interest in this matter and see that the members are provided with suitable accommodation, we think the Association would do well to meet according to its appointment.

The Executive Committee is carefully investigating the matter and will, in the near future, be able to decide as to the most feasible place for the meeting. We hope by the next issue of

THE REGISTER to be able to speak definitely on this subject. Wherever the meeting is held it is very desirable that there should be a large attendance not only of those who are members, but of those who will become members, and not at all excluding those who wish to come as visitors.

### The Annual Meeting of the National Association of Dental Faculties

Will be held at the time and place of the meeting of the National Dental Association, and it is very desirable that every member of the body should be present; that is, every college should be represented by at least one, or better, two delegates, each of whom should make special study of the various problems and questions that may come before the body for consideration.

Of course, all college men have these questions more or less in mind, or have some questions in mind which ought to receive more thorough consideration than they usually do before being brought up in its meetings.

Some subjects of more than usual importance will be presented for consideration and for action, and it is desirable that all should come prepared to take part in the adjustment of such questions.

Let all come with the determination to work for the elevation and best interests of the profession.

There will be several applications for membership. These should be carefully considered.

